# Finding trials for participants: an ethnographic study of successful recruitment strategies for clinical trials

**DOI:** 10.1186/s13063-025-08993-6

**Published:** 2025-08-08

**Authors:** Duncan J. Reynolds, Manuela Perrotta, Giuliano Maeilli

**Affiliations:** 1 Wolfson Institute of Population Health, Queen Mary University of London, London, UK; 2 School of Business and Management, Queen Mary University of London, London, UK

**Keywords:** Clinical trials, Recruitment, Trial matching, Novel strategy, Ethnography, Qualitative methods

## Abstract

**Background:**

Finding participants is one of the main barriers to the timely and successful running of clinical trials. Poor recruitment can lead to many issues, such as delays, which often leads to greater financial resources being put into the trials. We explore how a clinical trial centre with a good recruitment record is able to find participants for trials through successful innovative strategies.

**Methods:**

Ethnographic observations were conducted over a 17-month period at a clinical trial unit in London, UK, supplemented with semi-structured and ethnographic interviews with staff and participants. Observations focused on three hypertension clinical trials, including screening and pre-screening visits for them. Overall, the dataset included 353.5 h of observations, 322 min of semi-structures interviews, and ethnographic interviews not recorded.

**Results:**

It was found that The Centre engaged in innovative strategies by reversing the traditional logic of recruitment. As well as engaging in the traditional model of trial recruitment (1–4), where participants were found for specific trials (such as radio, newspaper, and social media adverts, and recruiting from other healthcare settings), The Centre reversed the logic and found trials for participants, a similar logic to how trial-matching platforms work. This was done by offering participants a range of trials when they first came to The Centre and offering other appropriate trials when they had finished the ones they were on. This reversed logic changed the nature of suitability of a participant to not only be someone eligible, but someone who staff would want and appreciate.

**Conclusion:**

Trouble recruiting for clinical trials is a perennial problem. Understanding how a successful centre recruits by reversing the logic of recruitment may allow other settings to attempt this to boost numbers and representativeness on trials. This opens up areas of future research to systematically test these methods.

## Introduction

Finding participants is one of the main barriers to the timely and successful running of clinical trials [1–3]. Recruitment can be difficult due to onerous exclusion criteria and high burden for trial participants [4], lack of interest [5], clinicians or family advising against participation [6], overoptimistic recruitment estimates [4], and more [4, 7–11]. Only 20% of clinical trials are completed on time, and difficulty in recruitment has been argued as a major reason for why [3, 12], with the average recruitment period being 4–5 years [13, 14]. The issue of recruitment has been highlighted by two of the UK’s largest funding bodies, the Medical Research Council (MRC) and the Health Technology Assessment Programme, who found that less than one-third of trials met their original recruitment targets [15]. Although more recent studies have shown some improvement, with figures between 40 and 50% [16–18], a better understanding of successful recruitment techniques is needed.

This is because the problem of poor recruitment can lead to many issues. Increasing the amount of time for recruitment often leads to greater financial resources being put into the trials [15]. Some estimates in the American pharmaceutical industry place delay costs at between $600,000 and $8,000,000 per day of delayed recruitment [19]. Further, there can be scientific issues with failing to recruit the number of participants desired. Not having enough participants can reduce the statistical power of a trial, with a potential increase in type II errors [17, 18]. Ramsey et al. [20] argue that many trials with hard clinical endpoints, such as death or hospitalisation, require many participants to be followed for a long period of time if they are to have enough statistical accuracy in answering the research questions which they pose. Linked to this, these delays in understanding the science could lead to delays in new treatments being made available to those who may benefit from them [14]. Further, issues in recruitment have also been argued to exacerbate health inequalities, for example, if there is a lack of diversity in clinical trials where certain demographic groups are under-represented [21]. There are also reported negative consequences for participants, as clinical trials expose participants to risk. Delays to trials may expose participants to harm when the overall trial is fruitless [14] and/or use up the goodwill of those who have joined [17].

Due to recruitment being a known problem with negative consequences, there have been attempts to increase rates of participation. New recruitment methods, such as social media targeting, have been shown to be effective in reaching certain demographics, such as males over the age of 55 [12, 22]. In their metareview of recruitment, Treweek et al. [15] investigated the impact of recruitment interventions, which they clustered into six categories: trial design, obtaining consent, approach to participants, financial incentives, training for recruiters, and trial coordination. Of these, there was shown to be some increase in recruitment associated with open trial design, telephone reminders, and paid participation in multiple studies. However, there was more evidence of little impact and even some of lower recruitment. Further critics of the effectiveness of recruitment interventions point to the fact that there are surprisingly few randomised evaluations of them [17]. Other work has proposed the use of artificial intelligence to aid with trial design, selection, and monitoring in an attempt to improve recruitment and retention outcomes [23]. Newington et al. [24] highlighted the problems of contacting people who may be willing to participate in relevant research in the future due to the consenting procedure. Currently, in the UK, the MRC and Health Research Association consent form templates do not include a question on whether participants are willing to be contacted in the future. This means that it is often not included, and potential participants are lost. In these cases, recruitment does not take place when there is potentially a pool of participants willing to be contacted about research opportunities. Perhaps as a result of the areas of difficulty, interest in the issue of recruitment has gone beyond the specific trials literature and has garnered the attention of social scientists and anthropologists, who have shown, for example, how financial incentives have led to healthy volunteers becoming “professional guinea pigs” [25, 26].

In recent years, there has been growth in “patient-centric” clinical trials in an attempt to improve recruitment [27–31]. This is defined as considering all the needs and concerns of patients, patient organisations, and regulators during the process of developing medical innovation [27, 30]. The logic being that if trials are able to fit more around patients’ lives, then recruitment and retention will improve. Analysis from The Economist indicated that trials which utilised a patient-centric approach to recruitment on average took 4 months to recruit 100 participants, compared with 7 months for those using other types of recruitment innovation [32]. As well as this, this method has been argued to aid the problem of a lack of recruitment in traditionally underserved communities into trials, as known barriers such as language, culture, and logistic-related issues can be focused on in trial design [27]. This has potential benefits societally as ethnic discrepancies and biases are known to affect clinical data, which can lead to risks of poor evidence of how drugs impact certain minority groups [7, 33, 34]. Specific methods for making trials “patient-centric” include involving patient and public groups in drafting consent forms, using technology to promote two-way communication between biopharma companies’ clinicians and patients, focusing on patient education, and building trust with potential participants [30].

The above methods of recruitment can all fall into the category of “finding participants for trials”, where the logic is that there are specific trials with specific inclusion and exclusion criteria, and people who fit these must be found. Potential participants are recruited for a specific trial, screened, and either consented onto the trial or rejected. Recent patient-centric approach to recruitment has in part flipped this logic, where trials are found for participants. This is seen in the growth of trial-matching platforms [35]. Here, potential participants can enter their health details and be provided with trials for which they can screen, meaning that they can decide themselves which trials are appropriate or not for their lives. Examples of these are NIHR’s Be Part of Research, Alzheimer’s Association TrialMatch®, Lilly Trials Connect, and Experimental Cancer Trial Finder by Cancer Research UK. These platforms often leverage AI to help participants find relevant trials [36]. This trial matching to potential participants shows the flipped logic of recruitment that this paper shall discuss in depth. Rather than clinical trials finding participants for trials, participants find trials for themselves, flipping the logic of finding participants for trials to finding trials for participants. Whilst these novel methods have the potential to aid clinical trial recruitment and representativeness [28], how the logic flipping (for example, through trial matching) can be implemented in practice by a clinical trial centre is not currently understood.

This paper is guided by the question: how does a clinical trial centre with a good record for recruitment find participants for trials through the flipping of the recruitment logic? We shall present evidence of how this approach was successfully implemented in a clinical trial centre in London, UK, with a good reputation and history of meeting recruitment targets. We shall show how this approach coexisted with traditional methods. This paper provides novel insights; first, we show how the finding participants for trials logic is implemented in a clinical trial centre as opposed to a charity or platforms. Second, we provide an in-depth analysis of how this recruitment strategy is achieved in practice by those working on and running clinical trials.

## Methods and setting

This study forms part of a larger PhD on the building of trust in clinical trials, where an ethnographic approach, informed by Heideggerian practice theory was utilised. More details on this can be found in Reynolds [37]. Ethnographic observations over a 17-month period were conducted, supplemented with interviews, at a UK clinical trial centre based in a diverse region of London. According to the 2021 census, the area in which The Centre was located had self-reported ethnicity of: 61.3% white, 14.1% Asian, and 10.5% black [38]. The staff and participants observed reflected this local diversity being from a range of nationalities and ethnicities. This centre was chosen because of its experience and success in running clinical trials. For example, it was the highest recruiting centre for a large, transnational, long-term, hypertension study. As a result, the local area has seen rapid decline in cardiovascular event rates, and an improvement of around 300% above the national average. The Centre ran trials on many areas of cardiovascular disease, as well as diabetes. For this study, three trials which investigated treatment for hypertension were observed. The Centre, trials, staff, and trial participants have all been anonymised as a condition of this study’s ethical approval. All the names of people and trials that follow are pseudonyms.

### The trials observed

All three trials observed were on different ways to manage hypertension. A summary of the trials can be seen below and in Table 1. These trials tested different interventions: combinations of already licensed drugs, the taking of an organic substance in drinking form, and an implanted device into a major artery. The trials were also a mix of commercially and non-commercially funded. This range was chosen in order to gain an understanding of trial practices across a range of trials within the same centre.

**Table 1:** Summary of clinical trials observed

Name of trial	Desired number of participants recruited from The Centre	Intervention being investigated	Funding	Length of trial for each participant
Snowdon	> 150	Combination of 4 different already licensed hypertension medications	NHS/UK Charity	24 weeks
Scafell Pike	< 100	Organic substance in juice form	Academic institution	16 weeks
Ben Nevis	10	Medical device inserted into a major artery during a surgical procedure	Private biotechnology company	2 years

#### Snowdon

The Snowdon trial aimed to determine if a person’s response to antihypertensive drugs differs by “self-defined” ethnicity, with the ultimate goal of being able to deliver personalised treatment for high blood pressure. Participants would take combinations of already licensed hypertension medications. The Centre planned to recruit high double figures of participants for this trial. A non-departmental government body and a charitable organisation jointly funded the trial. The trial was being undertaken at over 10 sites in the UK.

#### Ben Nevis

Ben Nevis was a commercial study sponsored by a private biotechnology company. It involved implanting a device into a major artery and delivering it intravascularly via a delivery catheter. The primary objective of the study was to evaluate the safety and effectiveness of the device in reducing blood pressure in participants with refractory hypertension. Up to 200 participants were being recruited at 50 different sites worldwide with single-figure recruitment targeted at The Centre. It was a sham-controlled trial.

#### Scafell Pike

Scafell Pike aimed to determine whether a natural, organic substance in juice form reduced over thickening of the heart muscle (left ventricular hypertrophy, LVH) and stiffness of the arteries when given to hypertensive participants. Overall, this trial aimed to recruit triple digit figures of participants at The Centre. A UK university and a UK hospital charity sponsored the trial. The study was double-blind, randomised, and placebo controlled.

All of the trials provided travel expenses. Ben Nevis and Scafell Pike also paid participants a nominal amount for their time (£150). All of the trials hoped to find ways to manage participants’ hypertension by optimising and/or reducing patients’ medication. When speaking to participants about motivation, reducing medication was frequently mentioned.

### Data collection

Observations occurred across the whole cycle of participant involvement with The Centre, including pre-screening, regular trial consultations, and post-trial visits, as well as observations of the running of The Centre from reception. All the observations were conducted by the first author, DJR. The majority of participants on the Snowdon trial were scheduled for Monday, Wednesday, and Friday mornings. For observations of this trial, DJR gained permission from the staff to arrive opportunistically and without prior scheduling for observations. Participants on the Scafell Pike and Ben Nevis trials were booked in at more varied times. For these observations, DJR would talk to the PIs at the start of the week about when visits were scheduled and specifically come to The Centre for them. All staff were consented to be observed by DJR at the start of the study. For trial participant consent, when participants arrived staff would ask them if they would mind having an ethnographer in the room during their visit. If they did not, DJR would enter, introduce himself and the study, and take written consent. DJR would then sit on a chair in the corner of the room. DJR took handwritten notes in a notebook during the consultations, before typing up fuller reflections at the end of the day, after observations had taken place. DJR’s placement and notetaking style were designed to be minimally disruptive to the running of the trials. Halfway through the time at The Centre, one of the clinical trial practitioners described DJR as “a piece of the furniture”. Reflecting on this, DJR believes that people got used to his presence over time and took it to mean that he was now immersed in The Centre (despite being overt).

During the observations, ethnographic interviews were also used when appropriate. These are informal, spontaneous interviews which take place during observations [39]. They allow the researcher to gain an understanding of what is going on during observation, from the point of view of those being observed. These took the form of clarifications and questions about what had been observed. Typically, on each trial visit there would be time when the staff would leave the room, for example, to collect study medication, or a piece of equipment needed for the next part of the consultation. DJR would often use this time to conduct the ethnographic interviews of the participants. These were informal and not recorded by Dictaphone. DJR would write down the responses in his notebook. When ethnographic interviews of staff occurred, they would happen after the participants had left at the end of the trial visit.

Healthcare professionals involved with the trials and some clinical trial participants were interviewed. On the Snowdon trial, two of the three clinical trial practitioners who worked on the trial were interviewed. By the time the interviews began the third was on maternity leave so not interviewed. The PI had been scheduled to be interviewed, but due to the COVID-19 pandemic, and the refocusing of staff towards this it was not deemed appropriate to add an additional burden to them in the form of an interview. For this same reason, neither of the two staff (one PI and one clinical trial practitioner) on the Ben Nevis trial were interviewed. The PI of Scafell Pike was interviewed as they were the person in charge of recruitment and the day-to-day running of the trial. This interview took place pre-pandemic lockdown. Participants on the trials were selected for interview based on their finishing of the trials at the planned time of the interviews commencing. Due to the COVID-19 pandemic, not as many interviews were conducted as had originally been planned with participants as it was decided not to invite participants for interview after the lockdown had been initiated, as all were classed as “vulnerable” and we did not want to add further demands to their lives at that stressful time. However, due to the ethnographic observation and ethnographic interview data previously gathered, we believe we had reached information power and a lower number of interviews did not detract from the work. Here, we understand information power [40]. At the end of the data collection period, it was believed that the additional data being collected was not revealing any meaningful new insights but rather repeating what had been seen previously. A summary of data collection can be seen in Table 2.

**Table 2:** Summary of data collection

Method	Details of data collection
Ethnographic observations	A total of 353.5 h of observation over a 17-month period was conducted. 151.25 h of observation was done of the Snowdon trial, 88.75 of Scafell Pike, 23.5 of Ben Nevis, 12 of participants on no specific trial, and 78 general observations from reception It is estimated that 20 staff members and 150 participants (or potential participants) were observed at The Centre
Semi-structured interviews	Overall, 332 min of interviews. Two clinical trial practitioners and two participants who had just completed the trial were interviewed from Snowdon. The PI and one recently finished participant from Scafell Pike were also interviewed. No one from Ben Nevis was interviewed. All the Snowdon interviews were conducted face-to-face. Both Scafell Pike interviews were conducted online using the Skype and Zoom platforms. Interviews length ranged from 42 to 62 min, with an average of 54 min
Ethnographic interviews	Approximately 30 ethnographic interviews were conducted. Due to time and practicality, they were not formally recorded but noted in DJR’s notebook. These took place every day of observation

### Data analysis

As DJR, a non-clinical social scientist, was eventually regarded by staff as “part of the furniture”, he recognised that this close rapport inevitably shaped what was noticed and how it was interpreted. To guard against over-identification, he kept reflexive fieldnotes and critically discussed emergent themes with co-authors, using his outsider disciplinary perspective to question taken-for-granted assumptions. The ethnography yielded 136 pages of fieldnotes along with 86 pages of transcripts from the interviews. These were thematically coded using NVIVO 12. Specifically, the data was analysed using thematic analysis, a qualitative technique for identifying and analysing themes was employed [41, 42]. Rather than counting words or phrases, this method allows for an in-depth analysis of the different areas identified in the data. The typing up of fieldnotes and the transcription of the interviews formed the first part of the analysis as it helped garner familiarisation with the data, and spot patterns and themes that had not noticed before. This took place as fieldwork was being conducted as DJR would type the handwritten fieldnotes onto a computer shortly after taking them, and interviews were transcribed within a week of their taking place. When formal data analysis started towards the end of the fieldwork, data was uploaded NVIVO 12. The data was read and reread, where initial codes were generated. These were then reviewed so that a cohesive story was being told. It must be noted that due to the large amount of ethnographic data that the story presented here is only one that could have been told, with others represented in other outputs. The data analysis was an iterative and reflexive process, with DJR repeatedly going back and rethinking and reworking codes after discussions with MP and GM. Codes were reduced or expanded and earlier coding revisited often as new insights were discussed and uncovered. Writing of findings is often seen as a stage that takes place after the analysis has been finished. However, the writing of findings is in itself a key part of analysis as it involves constantly returning to the data, reviewing the code, with the making new of connections.

## Study findings

### The traditional logic: finding participants for trials

The traditional logic of finding specific participants for specific clinical trials was observed at The Centre. These methods are common to many clinical trials [15], and at The Centre can be broken down into advertising (adverts in newspapers, on radio, and on social media) and recruitment from other medical settings (such as GPs and specialist hospital clinics). Both the Snowdon and Ben Nevis trials used newspaper, radio, and social media targeted adverts to attempt to attract participants. Although we did not gather specific data on how many participants were recruited by which method, from talking to participants, many said they became aware of The Centre and the trials because of these adverts, and this can be seen in the examples given in Vignettes 1 and 2.

**Table:** 

**Vignette 1—Ethnographic notes of Mr Windhoek** Mr Windhoek saw an advertisement for a clinical trial at The Centre in a newspaper. Although Mr Windhoek was not entirely certain if he met the criteria for participation in that specific trial, he was grappling with hypertension and was eager to explore options for addressing it. Upon contacting The Centre over the phone, it became evident that Mr Windhoek did not qualify for the trial mentioned in the newspaper. Nevertheless, the staff at The Centre invited him to come along for a visit, where they could do a blood pressure assessment, draw some blood, and discuss other ongoing trials. Their goal was to see if Mr Windhoek might be interested in screening for any of the other trials which they had going on at the end, even though he did not qualify for the one he had initially enquired about. When Mr Windhoek arrived at The Centre, he waited in the reception area before Dr Red called him into a consultation room. Before discussing Mr Windhoek’s health, Dr Red reached up and retrieved an A3-sized piece of paper from a shelf. This paper featured a hand-drawn image of the human body, specifically highlighting parts of the cardiovascular system and other organs, including the kidneys. The picture had lines drawn to all these different body parts with names of trials currently ongoing. These lines served as visual cues, indicating the part of the body that each trial aimed to impact. For example, the line for one trial led to the artery where the stent would be inserted, a line for a denervation trial pointed to the nerves it targeted, and the hypertension medication trial pointed to the heart. Using the diagram as a visual aid, Dr Red began a comprehensive walkthrough, carefully explaining each trial and its intended impact on hypertension. Mr Windhoek actively engaged in the conversation, interjecting several points to ask questions and seek clarification. Once Dr Red had covered all the trial options, he asked Mr Windhoek “Which of these sounds appealing to you?” Mr Windhoek expressed reservations about the denervation trial. Even though Dr Red described it as “minimally invasive”, he still did not like the sound of something “zapping” inside his body. Instead, he expressed a preference for the trial involving combinations of already licensed medications. This particular trial was light-heartedly referred to by Dr Red as the one that was “not known to give you green hair” because all the drugs used were already licensed with known side effects. With Mr Windhoek’s decision made, a screening visit for this trial was scheduled	

As well as running different types of adverts, The Centre frequently engaged in recruiting participants from other healthcare settings. This method was seen across all three observed trials and is a near-ubiquitous recruitment strategy across many UK clinical trials [14]. At The Centre, this was done in two main ways: through GP surgeries and through the local hospital hypertension clinic. First, when recruiting via GPs, the trials PI would approach local GP surgeries and ask them to send out a recruitment letter to all those in their registry who fit the trial criteria. The letters were trial-specific, but sometimes participants who came for one trial would end up on a different one. Dr Red often mentioned offhand that The Centre had a good relationship with local GP surgeries, having worked with them on previous trials. Second, the PIs of the Scafell Pike and Ben Nevis (Dr Indigo and Dr Blue) trials both worked for the hypertension clinic at the local tertiary hospital as well as at The Centre. This gave them extensive access to potential participants as all patients attending this clinic will have met the inclusion criteria (on all trials) of having high blood pressure (although they may not qualify for individual trials on other grounds). If either Dr Indigo or Dr Blue thought that someone would be a good participant (what this means shall be discussed further in the section “All within the context of who is suitable” and potential negatives of this in the discussion) for the trial, they would provide them with information on the trial and The Centre to see if they were interested. The above recruitment strategies all fall in line with the traditional logic of clinical trial recruitment, of finding participants for clinical trials with methods such as adverts and recruiting from other healthcare settings.

**Table:** 

**Vignette 2—Ethnographic notes Ms Rabat** Dr Red invited Ms Rabat into the consultation room. This marked Ms Rabat’s fourth visit to The Centre in the span of 2 weeks, making her somewhat of a regular visitor despite not being actively enrolled in any ongoing trials. Ms Rabat was initially asked to visit The Centre by Dr Indigo. Dr Indigo was not only her doctor who knew her from the hypertension clinic he held at the local NHS hospital, but also a PI on a trial at The Centre. Dr Indigo had identified Ms Rabat as a potential candidate for recruitment into one of the clinical trials conducted at The Centre, although he did not have a specific trial in mind for her. Instead, Ms Rabat was encouraged to visit The Centre, explore the available trials, and determine if any were of interest to her and if she met the eligibility criteria for participation. In my observations of his fourth visit to The Centre, Ms Rabat was undergoing the screening process for her third clinical trial. She had been deemed ineligible for the first two trials she had attempted to participate in, largely due to her mild hypertension and a combination of other long-term health conditions. These factors had caused her to “fail” the screening requirements for all three trials she had attempted to join. Regrettably for Ms Rabat, as she had continually expressed her desire to be on a trial, the outcome of this third screening was no different, and she was once again deemed ineligible. As Ms Rabat prepared to leave the consultation room, Dr Red expressed “We never got you into a trial, but we liked you so much we decided to keep you.” Despite the lack of success in securing a trial for Ms Rabat, The Centre made a commitment to stay in touch with her and promised to reach out in the future if any new trials that they deemed suitable and appropriate for her were initiated	

### An innovative strategy by reversing the logic: finding clinical trials for participants

In addition to traditional recruitment strategies, The Centre also actively sought to find trials for participants. What this means is that instead of finding participants who met the inclusion criteria (and did not meet the exclusion criteria) for specific trials, when people expressed an interest in a trial at The Centre, a lot of work was done to find a trial that was appropriate for by explaining a range of options to them. This was not a one-off action, but a strategy to aid recruitment. This shall be explored with the cases of how participants were offered trials and how The Centre recruited participants who were finishing other trials with them.

### Participants were offered a range of trials

On potential participants’ first visit to The Centre, the staff would explain the trials they had come to The Centre for and also inform them about the other research being done at the site. This is seen in Vignettes 1 and 2, where Mr Windhoek and Ms Rabat were invited in even though staff were not confident that they were eligible for the initial trial they had seen an advert for. Although participants were not eligible for their original trial of interest, the screening process was used as an opportunity to present other trials for which they might qualify. To do this, Mr Windhoek was presented with a picture of the human body, with arrows pointing to the areas upon which the trials acted. Dr Red and Mr Windhoek had a long conversation about the trials before Mr Windhoek chose one, for which he wished to screen. Ms Rabat was also invited for four screenings of different trials at The Centre. Here, the staff were repeatedly attempting to find a trial for a potential participant.

This recruitment strategy was also used when the participants were nearing the end of the trial they were currently on. Dr Red was observed casually mentioning to participants that they may have another trial at The Centre for which they might qualify. If the participants responded positively to this, more information was given, and the participant’s information would be passed on to the relevant clinician (with permission from the participant). This recruitment strategy of finding new trials for participants can be very clearly seen in the case of Mr Lisbon. Mr Lisbon originally came for a hypertension trial at The Centre, which was outside the scope of my observations. Once he completed that trial, he decided to join the Snowdon trial. Once he had completed Snowdon, Dr Red noted that he had a particular characteristic that the Scafell Pike trial was struggling to recruit participants with. He was asked if he would consider joining Scafell Pike, which he then did. Here, Mr Lisbon was recruited from another trial at The Centre twice and ended up doing three trials in a row. This recruitment strategy helped the smooth running of trials as the staff at The Centre had a long-running relationship with Mr Lisbon, and they could be confident that he would be a good trial participant (take his medications, turn up for appointments on time, etc.) because they already knew him from previous trials. This removed an element of risk involved with recruitment where participants may not be compliant in taking their medication. For the participants, a positive experience on the previous trials was an essential element to agreeing to stay at The Centre for future trials. For instance, in an ethnographic interview, Ms Maseru, another participant who was on her second trial at The Centre, commented that she was “made to feel very welcome” and joked that her experiences had turned her “into a trial junky!” By describing herself as a “trial junky”, Ms Maseru was showing her willingness to continue her relationship with The Centre by returning for multiple trials. Here, former participants were seen as a good place to recruit from as they had previously shown willingness to be engaged in a trial. Trials were then found for them so that they could remain at The Centre.

**Table:** 

**Vignette 3—Ethnographic notes Dr Blue and Mr Silver** Dr Blue and Mr Silver were seated at a vacant desk, located just outside the room where they typically held consultations for the Scafell Pike clinical trial. They were awaiting the arrival of the next participant, using the interim to talk about how the trial they were both working on was going. Their conversation inevitably drifted towards the challenges they were encountering in the recruitment process, particularly for the arm of the trial that required participants with left ventricular hypertrophy (LVH). As they discussed the topic further, Dr Blue and Mr Silver began to casually hypothesise the possible reasons behind the slow recruitment rate for this specific arm of the study. Dr Blue put forth his theory, suggesting that the reasons for the sluggish recruitment rate were primarily medical in nature. He elaborated that the trial’s exclusion criteria disqualified anyone with diabetes from participating. Given that a substantial number of individuals with LVH also had diabetes, they were instantly ruled out, thereby limiting the pool of eligible participants. In contrast, Mr Silver proposed an alternative theory. He argued that the slow recruitment rate could be attributed to the personality characteristics of the potential participants. He argued that individuals who had LVH as a condition might also be the kind of people who were less concerned about their overall health. If these individuals were less attentive to their health, they might also be less inclined to sign up for a clinical trial. The debate between Dr Blue and Mr Silver reached no definitive resolution. However, the conversation progressed towards brainstorming potential solutions to their recruitment predicament. Dr Blue mentioned that Dr Red was currently conducting a separate trial focusing on hypertensive patients, which involved participants undergoing an electrocardiogram (ECG). As ECGs are a diagnostic tool that can be used to identify LVH, Dr Blue and Mr Silver decided to approach Dr Red and ask him his help in flagging participants with LVH on his trial. Then, when they finished on Dr Red’s trial, Dr Blue and Mr Silver would approach these individuals and extend an invitation for them to join the Scafell Pike trial. Their rationale was that individuals who had already volunteered for a clinical trial would likely be more receptive to participating in another, compared to those with LVH who had never previously enrolled in a trial. Dr Red agreed to assist in their recruitment efforts, and he committed to marking the files of participants with LVH with red stickers, thereby making it easier for Dr Blue and Mr Silver to identify potential recruits for their study It was because of these stickers that Mr Lisbon was recruited for Scafell Pike. Mr Lisbon had been internally recruited for the Snowdon trial, having already completed one other trial at The Centre. Mr Lisbon had been on a different study at The Centre. During that study, it was noticed that he had hypertension, not something previously known by him or his GP. He was asked if he would consider taking part in a study on hypertension, which he agreed to do, which led to his involvement on Snowdon. During his final visit on the Snowdon trial, Dr Red noticed in his participant notes that his ECG had revealed that he had LVH. Dr Red knew that this was a characteristic of people that Dr Blue and his team were looking for the Scafell Pike trial. Dr Red mentioned to Mr Lisbon that they “stick around with us, we might not be done with you yet!” and that they had another trial which he could screen for, Scafell Pike. Mr Lisbon was warm to the idea and a screening visit was arranged for Mr Lisbon with Dr Blue, which was successful, and he joined his third trial at The Centre in a row	

### All within the context of who is suitable

From a medical and clinical trials perspective, suitability for a trial will be defined in terms of eligibility criteria. You cannot undertake recruitment of people for trials who are not eligible. However, in practice, suitability extends beyond mere eligibility; it encompasses attributes that render an individual cooperative and conducive to the successful conduct of the trial, thereby constituting a “good participant” (the potential positives and negatives of this shall be examined in the discussion). Examples of this happening can be seen above when participants who had finished one trial were asked to join another, such as Mr Lisbon in Vignette 3.

Whilst much of the literature has focused on the negative implications of inadequate or slow recruitment for clinical trials, what has been less well-spoken about is how non-suitable recruitment can lead to similar problems. If participants do not take their medication as required on the study, then this can negatively impact the scientific validity of the trial by potentially under- or over-estimating positive and negative effects. Non-adherence to taking medication is a well-known phenomenon in general with research in the UK suggesting that between 50 and 80% of people on hypertensive medications (outside clinical trial settings) are not taking them as they are prescribed [43]. There is evidence that participants in clinical trials are more compliant with their medication [44], and during an ethnographic interview Dr Red said their internal figures suggested 80% adherence. Similarly, if a participant is routinely late, or disruptive, this can lead to time delays in the running the trials which can negatively impact the experience of those in the trials as well as those working on them. In extreme cases, these time delays may have a negative financial impact in the same way as time delays due to slow recruitment. By reversing recruiting participants internally who had just finished another trial, staff could be more confident (although not certain) that they would be good participants, and would, for example, take their medication and arrive to appointments in a timely manner. This highlights the behavioural suitability of participants.

**Table:** 

**Vignette 4—Ethnographic notes Mr Berlin** On the morning of his final study visit, Mr Berlin arrived bearing a box of chocolates. Instead of pausing in the waiting room as was customary, he confidently walked through the healthcare practitioner’s open office door, where Ms Yellow, Ms Orange, and Ms Green were engaged in their preparatory work for the day. He approached them and presented the box of chocolates. Mr Berlin and the staff engaged in a friendly chat, exchanging pleasantries, although the box of chocolate was not opened. After a short while, Ms Orange made a comment that she had a lot of work to do, and Mr Berlin excused himself and returned to the waiting room, taking a seat to wait for his consultation to begin. Intrigued by his gesture, I asked Mr Berlin about his motivation for bringing the chocolates. He answered that, “it is my last visit, and they have given me something, so I thought I should give something in return.” Mr Berlin interpreted the study medication he had received from the staff as a gift, one that warranted reciprocation. Approximately 5 min later, Ms Orange called Mr Berlin into the consultation room for his appointment. As the session unfolded, I noticed a subtle change in Ms Orange’s approach with Mr Berlin compared to that I had seen with other participants. Contrary to her usual practice, she refrained from asking questions beyond those that were explicitly required by the study document. Her usual inquiries about participants’ day, their family, and their work were noticeably absent. This departure from her customary approach was quite striking, considering that in all the other consultations I had observed, Ms Orange had been more personable and inquisitive. Once the appointment concluded and Mr Berlin had exited The Centre for the final time, I took the opportunity to speak with Ms Orange. I was curious to understand why she had chosen not to make conversation about Mr Berlin’s life, as I had observed her do with others. Ms Orange explained that her decision was driven by practical considerations. She knew from experience that Mr Berlin enjoyed sharing his stories, and once he started, it could be challenging to steer the conversation back to the trial-related topics. Given that Mr Berlin was the first participant on her schedule for the day, Ms Orange was mindful of the potential impact on the rest of her and the participants’ days. She did not want to risk having her entire day delayed due to a prolonged consultation first thing in the morning. As a result, she opted to maintain a more focused and streamlined approach, sticking strictly to the questions required for the trial. Whilst this approach resulted in a consultation that was somewhat less friendly and warm than usual, it allowed her to ensure the appointment stayed on track and adhered to the necessary timeline	

The relationship between staff and participants was not always maintained after participation on trials had ended. Often, this was because The Centre had no other suitable trials to offer, but sometimes because staff did not see the participant as suitable (relationship-wise) for another trial. One example of this was Mr Berlin seen in Vignette 4. When Mr Berlin arrived for consultations, instead of waiting in the waiting room, he would always walk into the clinical trial practitioners’ office and start a conversation. Whilst staff did not seem to mind this, Ms Orange said she would rather he waited so she could finish the work she was doing in the office. Ms Orange also asked fewer personal questions during her consultations with Mr Berlin than with other participants. This was because she knew that Mr Berlin would often talk for a long time, meaning the consultation would overrun and cause delays. There were no overt or large-scale breakdowns in the relationship between staff and Mr Berlin, however when he was finished on the Snowdon trial, no effort was made to find him another one as staff found his personality and talkativeness slightly challenging to manage.

## Discussion

This paper has shown how a clinical trial centre with a strong record in recruitment complemented known recruitment strategies with innovative screening and retention strategies by flipping the traditional logic of finding participants for trials and engaged in the findings of trials for suitable participants. A consequence of this was that the nature of suitability to be a participant subtly changed. Not only did participants have to be eligible based upon the inclusion and exclusion criteria of the trials, but also be deemed suitable by staff. This may be in the context of not being disruptive, being likely to take medication as prescribed, and arriving to The Centre on time. Therefore, what is suitable is no longer purely medical, but also behavioural.

This reversing of the logic meant that novel recruitment practices were employed, with the potential to help recruitment numbers. A Cochrane Collaborate review into improving recruitment strategies in clinical trials (which looked at 45 studies and over 40,000 enrolled participants) did not identify this method of recruiting participants as a commonly used strategy [15]. More recent moves towards patient-centric trial and trial-matching recruitment methods have led to the introduction of platforms where potential participants can find suitable trials for themselves [36], but scholarly work had not previously focused on how clinical trials centres themselves reverse the logic and find trials for participants. In all of the cases in this paper, people showed an interest in joining a trial at The Centre, and staff attempted to establish a relationship with them by offering multiple screening visits for different trials in order to retain suitable participants. If The Centre had rejected potential participants who were not eligible then no relationship could be established, as they would not have seen each other again. Previous work has argued that opportunities for recruitment are often missed [45]. People already enrolled on a clinical trial, but nearing the end of their involvement present such an opportunity as their willingness to be involved in research is known, and their suitability largely known (recruitment is a risk as what if someone does not do what they are supposed to, for example, arrive on time and take their medication).

Flipping the logic of recruitment has the potential to be used as a strategy to improve the representation of traditionally underserved groups in clinical trials [27]. When a participant with such a background comes for a trial or completes one, then they could have other trials found for them, thus keeping them in research and helping to redress existing imbalances in who partake in trials. However, the potential for negative impacts of the reversed logic on representativeness must also be acknowledged. It is possible that staff may enact their own biases and see “suitable” or “good” participants as those who, for example, conform to their own cultural and social norms. In the findings, we showed how suitable participants were those who did not disrupt the running of the trials by, for example, talking too much. However, if recruitment takes place in a, for example, predominantly white and middle-class area then it is possible that these people will be prioritised for being offered more trials than those from underserved communities. Whilst we do not believe this was observed in our study, as the staff participants and location all had a high degree of diversity, this potential negative must be kept in mind and addressed so that it can be countered if needed.

### Strengths and weaknesses of the study

To our knowledge, this is the first study that identifies the flipped logic of clinical trial recruitment in practice being undertaken by a clinical trials centre. This study is indicative of how the strategy can be achieved in practice, but it is not an audit of its effectiveness. This provides an opportunity for other settings to adopt this practice in a way that suits the specific context in which they are situated. Previous research has identified stringent recruitment criteria as a barrier [4, 7]. By reversing the logic, and finding trials for participants, opportunities which would have been previously closed off to potential participants can be offered as if they fail the screening for one, they can be offered another. Further to this, previous research has shown that a lack of interest can cause recruitment problems [5], but offering a range of trials may help to find a trial which the participants are interested in and therefore more motivated to join.

One potential limitation to this method of recruitment which must be considered is that of recruiting only from a small pool of participants. As stated in the introduction, one persistent issue of poor recruitment can be an increase in health inequalities by under-representing certain communities [21]. Concentrating recruitment in a small cadre of repeat volunteers can inadvertently widen health inequities if the people invited to repeat trial participation are not from diverse backgrounds as there is a potential danger of increasing health inequalities by only testing on non-diverse groups of people. Those recruiting for trials using the reversed logic must be aware of this limitation and work to mitigate against it.

Criticism of ethnographic studies in healthcare have argued that they are not generalisable outside of the setting in which they are conducted [46, 47]. However, the interpretations of the findings offer useful ways of conceptualising outside of the specific context [48]. Employing these methods is important as it provides valuable insight into a specific location where recruitment is done [49]. We believe that the interpretation of the findings around the traditional and reversed logic will provide insight into other settings, as well as providing a novel method to be systematically tested in future research.

This study looked only at hypertension trials. All the trials observed in this study aimed to reduce or optimise medication for people with hypertension. As a result, when talking to participants, a common motivation was either themselves or people who would benefit from the research in the future having to take fewer medications. Offering potential participants a range of trials with similar goals is a possible reason for why the finding of trials for participants was able to work. To understand whether a similar approach would work where trials have different overall aims would require further studies.

This study employs a qualitative ethnographic approach, focusing on in situ observations and in-depth interviews to explore how the running of clinical trials in real-world settings. Whilst formal patient and public involvement (PPI) mechanisms were not used in the design of this study, participant perspectives remain central throughout. The ethnographic method is inherently responsive and participant-led, allowing individuals to shape the direction and emphasis of the research through ongoing engagement during data collection. Reflexive practice and opportunities for participants to discuss emerging findings were in place to ensure respectful and meaningful representation of participants’ views.

### Practical implications and future research

This study sheds light on the novel ways in which clinical trial recruitment can occur. The vast majority of existing literature highlights a traditional model of finding participants for trials. Future research can take the findings trials for participants approach identified here and further study how it can be achieved, and its effectiveness, in different contexts. How these practices need to be adapted for differing contexts provides a possible area of future study along with a systematic analysis of the effectiveness of reversing the logic.

## Data Availability

No data are available. This is because the ethics approval was granted based on the anonymity of individuals consenting to participate. Our participant information sheets and consent forms reflected this. No consent from research participants to make the raw data publicly available has been given.
